# *Enchytraeus crypticus* Avoid Soil Spiked with Microplastic

**DOI:** 10.3390/toxics8010010

**Published:** 2020-02-10

**Authors:** Stephan Pflugmacher, Johanna H. Huttunen, Marya-Anne von Wolff, Olli-Pekka Penttinen, Yong Jun Kim, Sanghun Kim, Simon M. Mitrovic, Maranda Esterhuizen-Londt

**Affiliations:** 1 Aquatic Ecotoxicology in an Urban Environment, Ecosystems and Environment Research Programme, Faculty of Biological and Environmental Sciences, University of Helsinki, Niemenkatu 73, 15140 Lahti, Finland; johanna.huttunen@helsinki.fi (J.H.H.); olli-pekka.penttinen@helsinki.fi (O.-P.P.); maranda.esterhuizen-londt@helsinki.fi (M.E.-L.); 2 Joint Laboratory of Applied Ecotoxicology, Environmental Safety Group, Korea Institute of Science and Technology Europe (KIST Europe) Forschungsgesellschaft mbH, Universität des Saarlandes Campus E7 1, 66123 Saarbrücken, Germanyyoungjunkim@kist-europe.de (Y.J.K.); 3 University of Helsinki, Helsinki Institute of Sustainability Science (HELSUS), Fabianinkatu 33, 00014 Helsinki, Finland; 4 Department of Civil Engineering, Group of Building Materials and Construction Chemistry, Technical University of Berlin, Gustav-Meyer-Allee 25, 13355 Berlin, Germany; 5 Department of Pharmaceutical Science and Technology, Centre for Chemical Safety Research, Kyungsung University, 309, Suyeong-ro, Nam-gu, Busan 48434, Korea; fatherofdamin@ks.ac.kr; 6 School of Life Sciences, University of Technology Sydney, Ultimo, NSW 2007, Australia; simon.mitrovic@uts.edu.au

**Keywords:** microplastic, *Enchytraeus crypticus*, enchytraeids, avoidance test, toxicity, oxidative stress, catalase, glutathione S-transferase

## Abstract

Microplastics (MPs) of varying sizes are widespread pollutants in our environment. The general opinion is that the smaller the size, the more dangerous the MPs are due to enhanced uptake possibilities. It would be of considerably ecological significance to understand the response of biota to microplastic contamination both physically and physiologically. Here, we report on an area choice experiment (avoidance test) using *Enchytraeus crypticus*, in which we mixed different amounts of high-density polyethylene microplastic particles into the soil. In all experimental scenarios, more Enchytraeids moved to the unspiked sections or chose a lower MP-concentration. Worms in contact with MP exhibited an enhanced oxidative stress status, measured as the induced activity of the antioxidative enzymes catalase and glutathione S-transferase. As plastic polymers per se are nontoxic, the exposure time employed was too short for chemicals to leach from the microplastic, and as the microplastic particles used in these experiments were too large (4 mm) to be consumed by the Enchytraeids, the likely cause for the avoidance and oxidative stress could be linked to altered soil properties.

## 1. Introduction

The contamination of terrestrial ecosystems and aquatic water bodies with plastics debris has become the so-called chemical footprint of our society. The European MSFD Working Group on Good Environmental Status (WG-GES) classifies plastic pollution as macroplastics (>25 mm), mesoplastics (5 to 25 mm), large microplastics (1 to 5 mm), and small microplastics below the 1 mm size [1]. Nevertheless, the particles do not remain static within these classification brackets, and due to weathering effects and mechanical actions, plastics will continue to degrade into microplastics (MPs) and further, as degradation does not reach a static end-point [2]. The smaller the particles are, the higher the uptake possibilities in organisms, plausibly even allowing the crossing of membranes [3].

Terrestrial ecosystems are on the forefront of MP contamination and are affected conceivably earlier than aquatic ecosystems [4,5]. Our cultivation industry not only uses plastic in the fields, but sludge from wastewater treatment plants that collect MP is used as fertilisers. Changes in soil structure and terrestrial geochemistry (water holding capacity, hydraulic conductivity, soil aggregation, and microbial activity) due to MP pollution have been demonstrated [4,5] which could, in turn, affect species distribution. This creates a toxic environment for the resident biota, which most concerns worms, which are essential in soil turnover and fertilisation. Other reported effects of MP in biota include internal damage due to the consumption or leaching of the additives contained in the plastics [6,7]. Amongst these additives are, e.g., bio-stabilisers, antimicrobials, antioxidants, antistatic agents, blowing agents, fillers/extenders, flame-retardants, fragrances, heat stabilisers, light stabilisers, pigments, and process aids [7]. The leached additives can accumulate in the soil, water, sediment, food, or even body tissues [8]. This could result in an ecosystem that causes severe adverse effects in the native biota. It would be of ecological importance, therefore, to understand if MP pollution could have an influence on the distribution of biota in an ecosystem, as this will contribute to the environmental crisis of decreasing biodiversity. If worm populations would avoid contaminated soils, this, in turn, would affect and alter the soil structure. Therefore, we investigated whether the distribution of biota could be affected by MP pollution in causing mortality or by migration. We selected the oligochaete *Enchytraeus crypticus* as a model organism, due to its abundance in soils globally, and as a likely candidate to be affected by the ubiquitous MP pollution. Enchytraeids are often used as model indicator organisms [9] for various kinds of chemical stressors in terrestrial ecosystems such as lindane, heavy metals, or phenmediphan [10,11,12]. The oligochaete *E. crypticus* was previously used to estimate the role of pH and PCB No 52 (2,2′,5,5′-Tetrachlorobiphenyl) as well as the effect of soil from former irrigation fields [13,14].

In the present study, mortality tests (using 0%, 2%, 4%, and 8% MP (*w*/*w*)) and avoidance tests (area choice test) were set up. For the avoidance tests, in each case, two choices of either soil void of MP or spiked with 2%, 4%, or 8% of MP (six combinations in total) were presented. The worms could move freely between the soil in two sections of the exposure vessels. To assess the responses of the worms to MP contamination in their environment, we used high-density polyethylene, as it is one of the most widespread plastic materials used today [15]. The MPs (4 mm particles) used were high-density polyethylene (HDPE) (confirmed by Fourier-transform infrared (FTIR) and Raman spectra) produced from threaded bottle caps—common trash seen globally. The MP type, size, and concentration were selected based on monitoring studies which reported on MP pollution in sediments, considering the most commonly detected MPs and their abundance and size distribution [16,17]. In addition, the size used (4 mm) was explicitly selected by selective sieving, so that the pieces would be too large to be consumed by the Enchytraeids. Thus, we could investigate effects other than consumption. We hypothesised that the worms would avoid MP-contaminated areas or would choose the lower MP concentration of the two options presented. After three days, the number of surviving worms in each section was evaluated, assessing mortality and distribution. Possible adverse effects on *E. crypticus* due to the roaming behaviour at a physiological level was assessed by determining the oxidative stress status measuring catalase and glutathione S-transferase activity as indicators.

## 2. Materials and Methods

### 2.1. Enchytraeus Crypticus Culture

*Enchytraeus crypticus* was continuously cultured at the University of Helsinki under the conditions outlined by Kobeticova et al. [18] and Castro-Ferreira et al. [9]. Briefly, the permanent culture of *E. crypticus* was maintained in the commercially available turf-free soil substrate (MeinWoody, Grub am Forst, Germany), pH 6–7, at a temperature of 18 ± 2 °C. The cultures were fed with oatmeal once a week by mixing the food into the soil substrate. Adults with a well-developed clitellum were used for the tests.

### 2.2. Microplastic

Plastic from new threaded bottle caps (green colour only) was used for all experiments. However, only caps with the Resin Identification Code (RIC) No. 2 or 02, indicating high-density polyethylene (HDPE)—one of the two most commonly used polymer types [19]—were selected. The caps were washed with tap water to remove possible adherent dirt or dust particles and dried at room temperature before shredding into MP. A desktop plastic recycler (SHR3D IT, 3devo B.V. Utrecht, Netherlands) with a sieve size of 4 mm was used to prepare MP granulate from threated bottle caps. To reach a homogenous granulates size of precisely 4 mm, the material was applied to the shredder five times and then sieved with a series of sieves (Test Sieve ASTM E11 containing steel oven wire) with a different mesh sizes to retain only the 4.00 mm particles (Endecotts Ltd, London, UK) (ISO 3310) and to achieve a homogenous MP sample material. During all stages, caution was taken not to self-contaminate the experimental set-up with other MP particles [20].

Confirmation of the type of the plastic from the bottle caps used was performed using Fourier-transform infrared spectroscopy (FTIR) on a PerkinElmer, Spectrum One (ATR-unit) for IR-spectra, using eight scans with a resolution of 4 cm^−1^ in a range of 4000–650 cm^−1^. Raman spectroscopy was applied as well using a Renishaw Invia Qontor confocal microscope at 785 nm, grating 1200 I/mm, exposure time 1s, 30 accumulations and 100% laser power, centre 1300 Raman shift/cm^−1^ and a 50-times objective.

### 2.3. Experimental Set-Up

The same turf-free soil (MeinWoody, Grub am Forst, Germany) used for cultivation was used for experimentation and consisted of 20% lingo fibres, 35% cocopeat washed, 10% spelt fermented, and 35% substrate compost. The soil had a pH ranging between 6 and 7 and was watered to 60% water holding capacity and kept at 18 ± 2 °C for a week.

**Figure 1:**
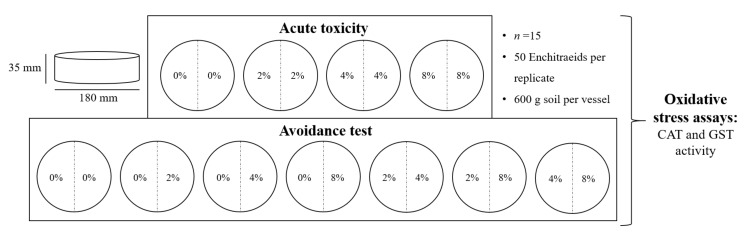
A schematic representation of the experimental setup. CAT: catalase; GST: glutathione S-transferase.

For the avoidance experiments, a modified protocol based on that described by Amorim et al. [21] was followed. Round paperboard forms with a diameter of 180 mm and a height of 35 mm were used as exposure vessels. To assess acute toxicity, the control vessels were filled with 600 g soil containing no MP (0%) or the respective MP concentration 2%, 4% or 8% mixed as homogeneously as possible (Figure 1). The exposure vessels were divided with a durable paper, adapted to the shape of the vessel, into two parts. For the avoidance tests, one-half was filled with 300 g of MP-free soil (0%) or either 2%, or 4% and the other half was filled with 300 g soil containing the respective MP percentage (2%, 4%, or 8%) (Figure 1).

The dividing paper was then removed, and the worms were placed in the middle of the exposure vessel; i.e., the contact line of both soil sides. The avoidance experiments were conducted for 72 h to allow the worms enough time to roam around and make their choice. As the exposure vessels used were larger than those reported by Amorim et al. [21], the longer exposure time was set based on extrapolation and preliminary tests, which assessed the time needed for the worms to travel the longer distances. When terminating the experiment, the soil was separated at the contact using a metal spatula, and the living worms were counted in the separated soil samples. The counted worms were shock-frozen in liquid nitrogen and stored at −80 °C until the extraction of the antioxidative stress enzymes. Mortality was assessed after 72 h of exposure to 0%, 2%, 4%, and 8% MP to assess acute toxicity.

### 2.4. Oxidative Stress

The worms from the avoidance tests were analysed to assess their oxidative stress status. Enzyme extracts were prepared by homogenising the worms in 0.1 M potassium phosphate buffer pH 6.5 containing 2.17 M glycerol, 1 mM ethylene-diamine-tetra acetic acid (EDTA), and 1.4 mM dithioerythritol (DTE). Cell debris was removed by centrifugation (10 min at 13,000 × g), and the supernatant was used for enzyme measurements [22]. Catalase activity (CAT, E.C. 1.11.1.6) was measured on a Tecan Infinite F Nano+ plate reader at 240 nm with the decrease of absorbance correlating to the disappearance of H_2_O_2_ [23]. The reaction mixture contained 50 mmol/L sodium phosphate buffer, 10 mmol/L H_2_O_2_ and 10 µL enzyme extract. The enzyme activity of CAT was defined as 1 mmol of H_2_O_2_ oxidised over 1 min at 25 °C and expressed as µkat/mg protein. Soluble (cytosolic) glutathione S-transferases (E.C. 2.5.1.18) were determined using the standard model substrate 1-chloro-2,4-dinitrobenzene (CDNB), according to Habig et al. [24]. Enzyme activities are given in katal per milligram protein (kat/mg protein); where katal (kat) is the conversion rate of one mol substrate per second. The protein content of each sample was determined according to the method of Bradford [25] using the Bradford protein-dye reagent (Sigma). Bovine serum albumin (98%, Sigma.Aldrich, St. Louis, Missouri, United States) was used as a standard protein for calibration of the assay method.

### 2.5. Statistical Analysis

SPSS software (IBM SPSS Statistics, Version 20) was used to perform a descriptive analysis based on the mean of the different endpoints chosen. Normality and homogeneity of variance were tested using Shapiro–Wilk and Levene’s test, respectively. When data proved to be normally and homogenously distributed, the data were submitted to the one-way analysis of variance (ANOVA) followed by Tukey post hoc in SPSS. When tests for normality and homogeneity were not satisfied, the non-parametric Mann–Whitney-U Test together with pairwise comparisons was employed. We set the alpha value as 0.05 level for significance [26]. All data are graphically expressed as mean ± standard deviation (SD).

## 3. Results and Discussion

### 3.1. Toxicity and Avoidance Tests

**Figure A1:**
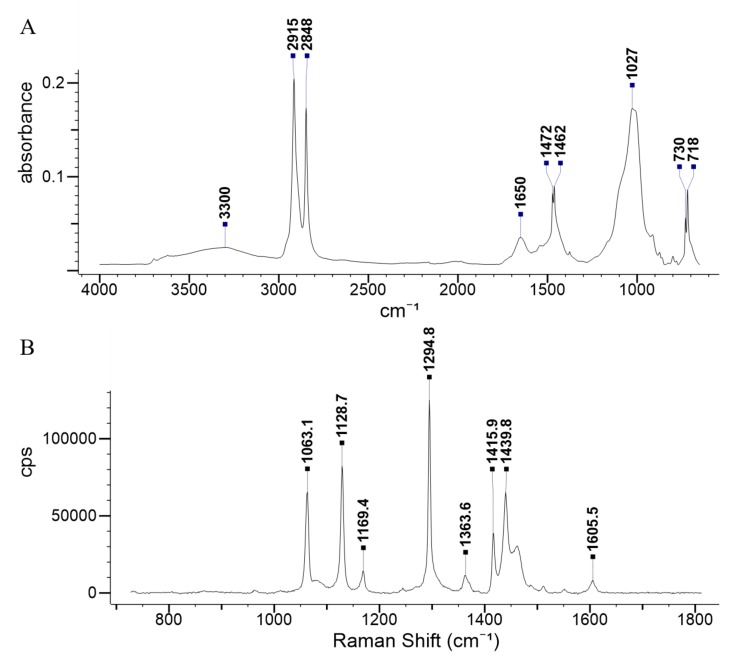
Confirmation of the MP type as high-density polyethylene (HDPE) with (**A**) Fourier transform infrared (FTIR) spectra and (**B**) Raman spectra.

An avoidance test is defined as an organism’s active selection between two samples exhibiting different properties [27]. Therefore, in the present study, *Enchytraeus* sp. worms were placed in exposure vessels containing two non-obstructed halves, which consisted of soil mixed with different percentages of MP ranging from 0% to 8% (*w*/*w*) to determine their preference, if any. FTIR and Raman spectroscopic analyses of the bottle cap plastics confirmed that they were indeed HDPE (Figure A1). To assess the acute toxicity (mortality), the two halves consisted of the same MP percentages; i.e., with no MP on both sides, or 2%, 4%, or 8% on both sides, respectively. In all these mortality tests, where the exposure percentages were equal throughout the exposure vessel, *E. crypticus* distributed equally between the two halves (Table 1).

An increased percentage of HDPE MP in the soil (0% to 8%) resulted in the *E. crypticus* mortality increasing from 2% to 14% (Table 1). However, the properties of the monomer ethylene used to produce HDPE were previously reported not to cause toxicity nor to exhibit estrogenic activity [28]. Consumption leading to internal obstruction and damage is unlikely due to the size of the particles used. However, due to the manufacturing process, all plastics can contain residual chemicals, including catalysts necessary for the polymerisation reactions, which could quickly leach from new plastics. Additives such as stabilisers, UV-blockers, plasticisers, antioxidants, and colourants are added to the plastic formulation to provide the final product with the necessary properties. These additives are retained in the plastic bound to the polymer matrix through van der Waals forces [29]. The leaching of those chemicals due to the breakage of these weak bonds during the degradation of plastics might therefore occur [7,30] and affect our environment [28,31]. The toxicity observed here could be due to leaching additives from the shredded bottle caps [4,5]. However, it is more likely that the MP particles could have caused changes in the soil structure [4,5], which resulted in undesirable conditions for the Enchytraeids [21].

**Figure 2:**
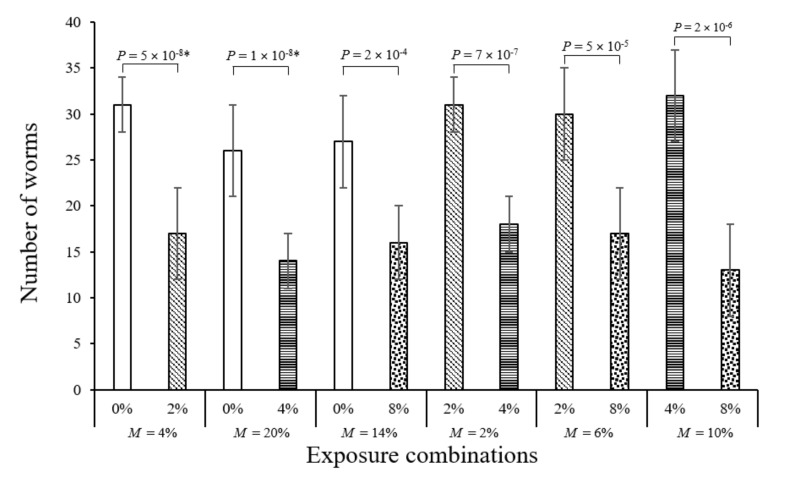
Worms counted in non-spiked and MP-spiked areas of the avoidance test vessel. Data represent mean worm count per area ± standard deviation (*n* = 15, at 50 worms per independent replicate). The average percentage mortality per pairing is given under each section as *M*. Differences between the treatments were tested by one-way ANOVA and Tukey post hoc when the data were normally and homogeneously distributed. When data were not homogenously distributed, even after transformation, the non-parametric Kruskal–Wallis test with pairwise comparisons was used.

In all avoidance test pairings, where non-spiked soil was presented against MP spiked soil (0% to 2%, 0% to 4%, and 0% to 8%), more *E. crypticus* (avg. 60% ± 4%) moved to the non-spiked half (Figure 2). The Enchitraeids’ preference was higher by factors of 1.6 to 1.8 in favour of the non-spiked side. The average survival rates in the pairings with an unspiked side ranged from 80% to 96%. Following the experimental set-up from Kerekes and Feigl [32], all MP concentrations were paired against each other for the avoidance tests; i.e., 2% to 4%, 2% to 8%, and 4% to 8%. In these pairings offering a lower and a higher MP concentration as a choice, *E. crypticus* also preferred the lower MP concentration in all pairings (Figure 2). The worms favoured the lower MP concentration side by factors of 1.7 to 2.7 with increasing MP percentages. Thus, in the avoidance tests, Enchytraeids showed a clear preference for the MP-free sides or less polluted sides, most likely due to altered soil properties, such as decreased bulk soil density and decreased microbial activity [5] as the MP particles were too large to consume. The possibility of leaching cannot be completely excluded as a potential reason for the avoidance; however, this is unlikely as the exposure time was too short for leaching and exposure was carried out at room temperature (18 ± 2 °C) and not under solar irradiation [33,34,35].

### 3.2. Oxidative Stress

**Figure 3:**
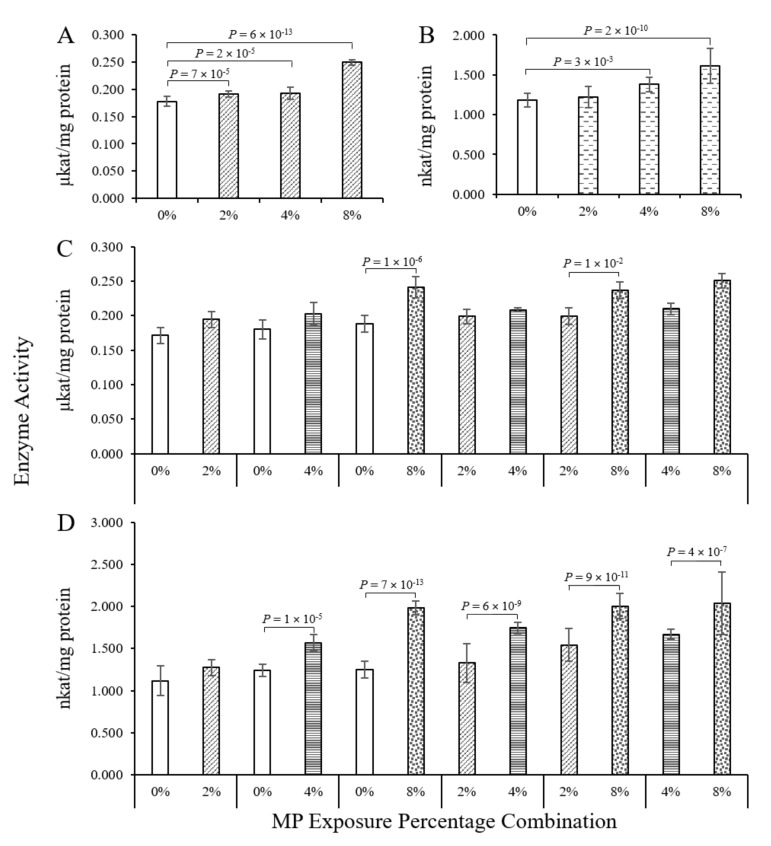
(**A**) Catalase activity in the 0% MP on both sides pairing, as well as in pairings with the same MP concentration on both sides. (**B**) GST activity in a 0% to 0% pairing, as well as in pairings with the same MP percentage on both sides. Data represent mean enzyme activity ± standard deviation (*n* = 9, at 50 worms per independent replicate). (**C**) Catalase activity in worms from soil containing 0% to 2%; 4%, and 8% MP, as well as worms from the avoidance experiment from clean and MP-spiked sides of different MP concentrations. (**D**) Glutathione S-transferase activity in control worms from soil containing 2%, 4% and 8% MP, as well as worms from the avoidance experiment from clean and MP-spiked sides of different MP concentrations. Data represent mean enzyme activity ± standard deviation (*n* = 3, at 50 worms per independent replicate). When data were not homogenously distributed, even after transformation, the non-parametric Kruskal–Wallis test with pairwise comparisons were used.

As exposure to MP is correlated to oxidative stress [36], we measured the activity of catalase (CAT) and glutathione S-transferase (GST) in the worms after exposure to different HDPE MP percentages in soil. Exposure to the HDPE MP caused the CAT activity of the Enchytraeids to increase dose-dependently (Figure 3A). In the pairings consisting of 0% to 8% as well as 2% to 8% MP, the worms exhibited higher CAT activity (Figure 3C), suggesting that 8% (*w*/*w*) MP in soil induced oxidative stress in these worms.

GST is known as a biotransformation enzyme; however, it is also involved in the antioxidative stress defence as it metabolises end-products such as malondialdehyde and 4-hydroxynonenal derived from lipid-peroxidation [22]. As with CAT, the increasing HDPE MP percentages in the soil resulted in a dose-dependent increase of the GST enzyme activity in the Enchytraeids (Figure 3B). All pairings, except 0% to 2% HDPE MP, resulted in elevated GST activity (Figure 3D).

In most of the cases presented here, exposure to MP in the soil led to an increase in enzyme activity, indicating the elicitation of an antioxidative stress response. For nanoparticles and microbeads with a size range between 0.05 and 6 µm, it is known that the toxicity is closely correlated to the uptake into organisms and the generation of reactive oxygen species (ROS) [36,37,38]. An increase of ROS will lead to oxidative-stress-induced signalling pathways. However, the MP particles used here were specifically selected to have a size of 4 mm; therefore, they are too large to be taken up by the oligochaetes used. As leaching is implausible, altered soil properties may have induced oxidative stress in agreement with the findings reported by Howcroft et al. [39].

In conclusion, the results show that the oligochaetes preferred an MP-free environment and that, in the presence of MP, their antioxidative stress response was elevated. As uptake and leaching under the experimental conditions used here are unlikely, altered soil properties due to the presence of MP may be the cause for the results observed. More research is needed to investigate long-time exposure and the toxicity of the compounds leaching from MP in our environment to better understand the adverse effects of MP in our ecosystems. This is the first study to show an area choice test for Enchytraeids avoiding MP spiked sites.

## Figures and Tables

**Table 1:** Acute toxicity and area preference in pairings with the same percentage of microplastic (MP) in both halves (*n* = 15, at 50 worms per independent replicate). Significance was tested by pairwise t-tests after normality and homogeneity tests were satisfied.

%MP in Soil Halves (*w*/*w*)	Worms in	Worms out	Mortality	Distribution in the Two Exposure Vessel Halves		Distribution Comparison (*P*-Value)
0%	50 ± 0	49 ± 1	2%	25 ± 3	24 ± 3	0.328
2%	50 ± 0	46 ± 2	8%	23 ± 2	23 ± 3	0.618
4%	50 ± 0	45 ± 1	10%	23 ± 3	22 ± 2	0.186
8%	50 ± 0	43 ± 1	14%	22 ± 2	21 ± 2	0.340
